# NIGROSTRIATAL DOPAMINE AND PHYSICAL PERFORMANCE FATIGABILITY IN COMMUNITY-DWELLING OLDER ADULTS

**DOI:** 10.1093/geroni/igae098.0238

**Published:** 2024-12-31

**Authors:** Caterina Rosano, Emma Gay, Paul Coen, Andrea Rosso, Anne Newman, Stephen Kritchevsky, Nancy Glynn

**Affiliations:** University of Pittsburgh, Pittsburgh, Pennsylvania, United States; University of Pittsburgh School of Public Health, Pittsburgh, Pennsylvania, United States; AdventHealth, Orlando, Florida, United States; University of Pittsburgh, Pittsburgh, Pennsylvania, United States; University of Pittsburgh, University of Pittsburgh, Pennsylvania, United States; Wake Forest University School of Medicine, Wake Forest University School of Medicine, North Carolina, United States; University of Pittsburgh School of Public Health, Pittsburgh, Pennsylvania, United States

## Abstract

Increasing nigrostriatal dopaminergic signaling can ameliorate performance fatigability in healthy young adults, but its role in community-dwelling older adults is not known. We hypothesized higher nigrostriatal dopaminergic integrity would be associated with lower performance fatigability, independent of other factors that impact physical performance fatigability, including cardiorespiratory fitness and muscle energetics. In 125 participants of the Study of Muscle, Mobility and Aging (SOMMA), performance fatigability was measured as performance deterioration during a fast-paced 400 meter walk (% slowing down from the 2nd to the 9th lap, higher = more performance deterioration). Nigrostriatal DA integrity was measured using (+)-[11C]dihydrotetrabenazine (DTBZ) PET imaging. The binding signal was obtained separately for the subregions regulating sensorimotor (posterior putamen), reward (ventral striatum) and executive control processes (dorsal striatum). Multivariable linear regression models of performance fatigability (dependent variable) estimated the coefficients of dopamine integrity in striatal subregions (in separate models), adjusted for demographics, cognition, mitochondrial respiration (assayed in muscle biopsies) and cardiorespiratory fitness (via cardiopulmonary exercise testing). In this sample (mean age 75.4±4.0 years, 59% women), mean performance deterioration was 2.5%±4.7%. Higher [11C]-DTBZ binding in the posterior putamen was significantly associated with lower performance fatigability (demographic-adjusted standardized Beta= -1.18, 95% CI:-2.11, -0.25); results remained independent of further adjustment for other covariates, including cognition, cardiorespiratory fitness, and muscle energetics (standardized Beta= -1.03, 95% CI:-2.02, -0.10). Association with other striatal subregions were not significant. Dopaminergic integrity in the sensorimotor striatum may influence performance fatigability in older adults regardless of clinically overt diseases, and independent of other aging systems.

